# Mutational profile of primary breast diffuse large B-cell lymphoma

**DOI:** 10.18632/oncotarget.21986

**Published:** 2017-10-24

**Authors:** Fernando Franco, Julia González-Rincón, Javier Lavernia, Juan F. García, Paloma Martín, Carmen Bellas, Miguel A. Piris, Lucia Pedrosa, José Miramón, José Gómez-Codina, Delvys Rodríguez-Abreu, Isidro Machado, Carmen Illueca, Jesús Alfaro, Mariano Provencio, Margarita Sánchez-Beato

**Affiliations:** ^1^ Medical Oncology Department, Hospital Universitario Puerta de Hierro, Madrid, Spain; ^2^ GOTEL (Spanish Lymphoma Oncology Group), Madrid, Spain; ^3^ Group of Research in Lymphomas, Medical Oncology Department, Instituto de Investigación Sanitaria Puerta de Hierro-Segovia de Arana, Madrid, Spain; ^4^ Centro de Investigación Biomédica en Red de Cáncer (CIBERONC), Madrid, Spain; ^5^ Medical Oncology Department, Instituto Valenciano de Oncología, Valencia, Spain; ^6^ Pathology Department, MD Anderson Cancer Center, Madrid, Spain; ^7^ Pathology Department, Hospital Universitario Puerta de Hierro, Madrid, Spain; ^8^ Pathology Department, Hospital Universitario Fundación Jiménez Díaz, Madrid, Spain; ^9^ Medical Oncology Department, Hospital Serranía de Ronda, Málaga, Spain; ^10^ Medical Oncology Department, Hospital Universitari i Politècnic La Fe, Valencia, Spain; ^11^ Medical Oncology Department, Hospital Universitario Insular de Gran Canaria, Las Palmas, Spain; ^12^ Pathology Department, Instituto Valenciano de Oncología, Valencia, Spain; ^13^ Medical Oncology Department, Instituto Oncológico de Kutxa, Donostia, Spain

**Keywords:** primary breast lymphoma, diffuse large B-cell lymphoma, cell of origin, NFkB pathway, PIM1

## Abstract

Primary breast lymphoma is a rare form of extra-nodal lymphoid neoplasm. The most common histological type is the diffuse large B-cell lymphoma, which represents 60–80% of all the cases. Our study analyzes the mutational profile of the primary lymphoma of the breast through targeted massive sequencing with a panel of 38 genes in a group of 17 patients with primary breast diffuse large B-cell lymphoma. Seventy-point-five percent of the patients presented with stage IE and 29.5% with stage IIE. 44% of the cases correspond to lymphomas with germinal center phenotype and 33.3% to activated B-cell. The genes with a higher mutational frequency include *PIM1* (in 50% of the analyzed samples), *MYD88* (39%), *CD79B, PRDM1* and *CARD11* (17%), *KMT2D*, *TNFIAP3* and *CREBBP* (11%). The profile of mutant genes involves mostly the NFκB signaling pathway. The high frequency of mutations in *PIM1* compared with other lymphomas may have implications in the clinical presentation and evolution of this type of lymphoma.

## INTRODUCTION

Primary breast lymphoma (PBL) is a rare form of extra-nodal lymphoid neoplasm. It represents 1% of non-Hodgkin’s lymphomas (NHL) and 2,2% of extra-nodal lymphomas. PBL usually presents as a painless mass of progressive growth with or without ipsilateral axillary lymph nodes and more than 96% of the cases affect to women [1–3]. It was first described in 1972 by Wiseman and Liao [4], who defined diagnostic criteria, which would be later modified by Hugh et al [5], defining the PBL as the infiltration of breast tissue by lymphoma with or without regional lymph node in patients without a history of prior nodal or extra-nodal lymphoma and systemic disease at the time of diagnosis. More than 95% of the cases correspond to B-type NHL and the most common histopathological type is diffuse large B-cell lymphoma (DLBCL), which represents 60–80% of all the cases. Other less frequent subtypes include follicular lymphoma and marginal zone lymphoma MALT-type [5–6]. The treatment of primary breast DLBCL (PB-DLBCL) is based on regimes like R-CHOP (Cyclophosphamide, Doxorrubicin, Vincristine, Prednisone and Rituximab) or similar treatments. In the Rituximab era an overall survival (OS) at 5 years may reach 87% [6–9]. The genetic alterations of PB-DLBCL have not been previously analyzed, except for the study from Taniguchi et al regarding MYD88 and CD79B [10], partly due to the infrequency of this presentation and the difficulty in obtaining sufficient tumor tissue to perform molecular analysis. Our objective is, therefore, to contribute to the knowledge on the mutational landscape of PB-DLBCL.

## RESULTS

The study population consists of 17 patients, all women, with PB-DLBCL diagnosed between 1993 and 2016 in different medical centers in Spain. The average age of diagnosis was 66 years (range, 29–84) (Table 1). In nine cases, the left breast was involved; in 7 cases the right one and both in one case. Seventy-point-five percent of the patients presented with stage IE and 29.5% stage IIE. All patients received immunochemotherapy (R-CHOP or similar), except one who was treated with CHOP. All the patients achieved complete response (CR) (Table1). There were 2 relapses in the contralateral breast, both treated with second-line regimens that achieved CR. From one of these patients we confirmed the diagnosis and acquired the relapsing samples. There were also 1 systemic nodal relapse and 1 brain relapse, both of which died from disease progression. The median progression free survival (PFS) was 7 years and the median OS was 16 years.

**Table 1:** Clinical data of PB-DLBCL patients

Patient	Age	Laterality	Nodal involvement	Stage	IPI	Chemo-therapy	Cycles	Radio-therapy	Response to treatment	Relapse	Treatment	Time to progression	Response to treatment
01	84	Left	No	IE	2	R-CVP	5	Yes	CR				
02	78	Right	No	IE	1	R-CHOP	4	No	CR				
03	74	Left	No	IE	2	R-CHOP	4	No	CR				
04	40	Right	Yes	IIE	0	R-CHOP	4	Yes	CR				
05	60	Left	No	IE	1	R-CHOP	8	No	CR				
06	29	Right	No	IE	0	R-CHOP	6	No	CR				
07	78	Bilateral	Yes	IIE	1	R-CHOP	6	No	CR				
08	73	Left	No	IE	2	CHOP	6	No	CR				
09	75	Left	No	IE	1	R-CHOP	4	No	CR				
10	79	Left	No	IE	2	R-CHOP	6	No	CR				
11	83	Right	Yes	IIE	2	R-MVP	6	No	CR	Nodal	No treatment	7 m	Progression
12	70	Right	No	IE	1	R-CHOP	6	No	CR	Contralateral breast	R-ESHAP	80 m	CR
13	79	Left	No	IE	1	R-CHOP	4	Yes	CR				
14	62	Right	No	IE	1	R-CHOP	4	Yes	CR	Brain	Methotrexate	23 m	Progression
15	70	Left	No	IE	2	R-CHOP	6	No	CR				
16	46	Left	Yes	IIE	ND	R-CHOP	6	No	CR				
17	42	Right	Yes	IIE	ND	R-CHOP	6	Yes	CR	Contralateral breast	R-COMP	157 m	CR

Abbreviations: R-CVP: Rituximab, Cyclophosphamide, Vincristine, Prednisone; R-CHOP: Rituximab, Cyclophosphamide, Doxorubicin, Prednisone; R-MVP: Rituximab, Methotrexate, Vincristine, Procarbazine; R-COMP: Rituximab, Cyclophosphamide, Myocet, Prednisone; R-ESHAP: Rituximab, Etoposide, Methylprednisolone, Cytarabine, Cisplatin; CR: Complete response; ND: No data; m: months

Forty-four-point-four percent of the analyzed samples were classified, according to Hans’s algorithm (see Materials and Methods), as germinal center B-cell (GCB) DLBCL, 33.3% as non-GCB, while 16.6% were unclassified (Table 2). The proliferation index, determined by Ki-67 expression, was higher than or equal to 80% in 16/18 samples; only samples from patients 2 and 3 showed values of Ki67 expression of 57 and 70%, respectively.

**Table 2:** Immunohistochemical results and cell of origin (COO) determined according to Hans’s algorithm in PB-DLBCL

SAMPLE	CD10	CD20	BCL6	BCL2	MUM1	Ki67	COO
01	+	+	+	+	ND	96%	GCB
03	–	+	+	+	–	70%	GCB
04	+	+	+	–	–	80%	GCB
05	+	+	+	+	ND	80%	GCB
06	ND	+	+	+	–	80%	GCB
07	ND	+	+	+	–	90%	GCB
08	–	+	+	+	–	80%	GCB
09	–	+	+	+	–	85%	GCB
02	–	+	–	+	ND	59%	Non-GCB
10	–	+	+	+	+	80%	Non-GCB
11	–	+	–	–	+	100%	Non-GCB
12a	ND	+	ND	ND	ND	98%	ND
12b	–	+	+	+	+	98%	Non-GCB
15	ND	+	+	+	+	80%	Non-GCB
16	–	+	+	+	+	80%	Non-GCB
17	–	+	+	+	+	80%	Non-GCB
13	ND	+	ND	ND	ND	90%	ND
14	–	+	+	+	ND	100%	ND

GCB: germinal center B-cell; non-GCB: non-germinal center B-cell, ND: No data.

**Figure 1:**
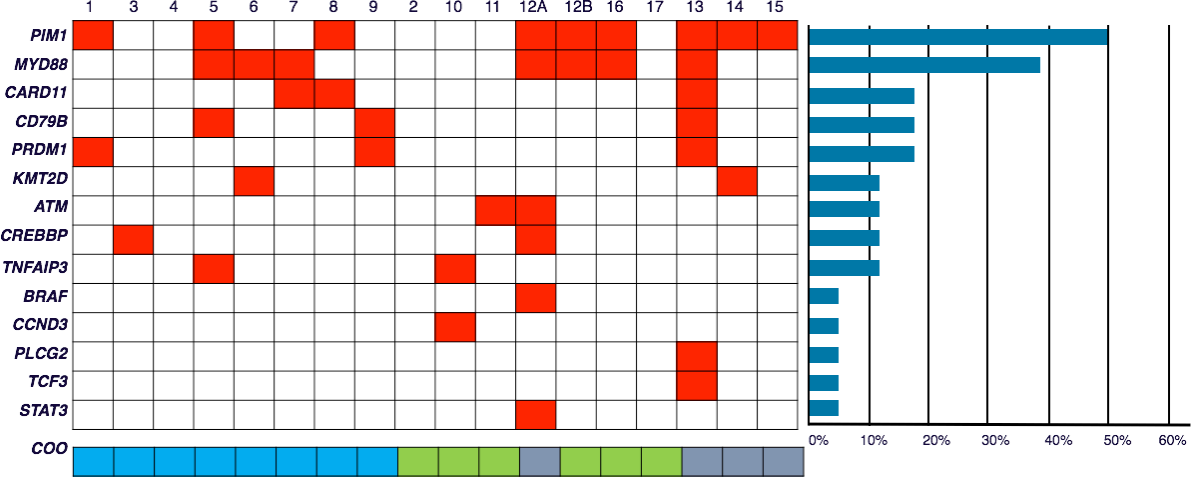
Frequencies and distribution of gene mutations in PB-DLBCL, and cell of origin (CCO) A total of 18 PB-DLBCL samples were subjected to targeted massive paralleled sequencing for 38 frequently mutated genes in DLBCL. Columns represent samples and the rows represent the mutated genes. Colored box (red) indicates at least 1 mutation in the given gene. The bottom row shows the COO for each sample (blue: GCB; green: non-GCB; grey: no data). Patient number 12 had two samples (12a, taken at the time of diagnosis, and 12b at the time of relapse). The horizontal bars at the right of the figure represents percentage of mutated samples.

We designed a targeted sequencing panel for aggressive B-cell lymphomas including 38 recurrently mutated genes described in the literature (Supplementary Table 1) [11–14]. TruSeq^®^ Custom Amplicon Low Input Library for dual-strand sequencing (Illumina Inc. San Diego CA, USA) was used. Median coverage for the amplicons for pool A was 379× (50–1098×) and pool B was 426× (30–2149×). After sequencing 18 samples from the 17 patients included in the study, we found a total of 52 non-synonimous and 21 synonimous mutations; at least 1 mutated gene (taking into account exclusively missense, nonsense, frameshift and splicing mutations) were found in 15 of them. Samples 5, 12a and 13 had the highest numbers of mutated genes, with 4, 6 and 7 respectively (Figure 1, Table 3 and Supplementary Table 2). Some cases had more than one mutation per gene. We identified mutations in 14 of the 38 selected genes (*PIM1, MYD88, KMT2D, CARD11, CD79B, PRDM1, ATM, BRAF, CREBBP, TNFAIP3, CCND3, PLCG2, TCF3* and *STAT3*) (Figure 1, Table 3 and Supplementary Table 2). The recurrently mutated genes were *PIM1* (in 9/18 samples; 50%), *MYD88* (7/18, 39%), *CD79B, PRDM1* and *CARD11* (3/18; 17%), *KMT2D, TNFAIP3* and *CREBBP* (2/18; 11%) (Figure 1, Table 3 and Supplementary Tables 2 and 3).

**Table 3:** Non-synonymous somatic variants identified in PB-DLBCL samples

Sample	Position	Ref/Alt	Gene	Function	Exon	CDS change	aa change
1	6:37140780	G/A	PIM1	missense	exon5	c.G616A	p.V206M
	6:106536093	C/CTCCA	PRDM1	frameshift_variant	exon2	c.60_61insCCGGCT	p.Ser21fs
3	16:3820828	G/A	CREBBP	missense	exon14	c.C2623T	p.P875S
5	17:62006799	A/G	CD79B	missense	exon5	c.T586C	p.Y196H
	3:38182641	T/C	MYD88	missense	exon5	c.T794C	p.L265P
	6:37138405	C/A	PIM1	missense	exon1	c.C54A	p.N18K
	6:37138427	G/A	PIM1	missense	exon1	c.G76A	p.A26T
	6:37139097	G/A	PIM1	missense	exon4	c.G437A	p.S146N
	6:37139180	C/T	PIM1	missense	exon4	c.C520T	p.L174F
	6:138200190	C/A	TNFAIP3	stopgain	exon7	c.C1608A	p.C536X
6	12:49420433	G/A	KMT2D	missense	exon48	c.C15316T	p.R5106C
	3:38182641	T/C	MYD88	missense	exon5	c.T794C	p.L265P
7	7:2978320	C/T	CARD11	missense	exon7	c.G1010A	p.R337Q
	3:38182641	T/C	MYD88	missense	exon5	c.T794C	p.L265P
8	7:2987341	G/A	CARD11	missense	exon3	c.C88T	p.R30W
	6:37139033	C/T	PIM1	missense	exon4	c.C373T	p.P125S
9	17:62006797	G/C	CD79B	stopgain	exon5	c.C588G	p.Y196X
	6:106536235	G/A	PRDM1	missense	exon2	c.G202A	p.D68N
10	6:41903731	G/A	CCND3	stopgain	exon5	c.C826T	p.Q276X
	6:138200458	C/CT	TNFAIP3	frameshift_variant	exon7	c.1876_1877insT	p.Leu626fs
11	11:108172446	G/A	ATM	stopgain	exon35	c.G5249A	p.W1750X
12a	11:108119789	C/T	ATM	missense	exon9	c.C1195T	p.H399Y
	7:140482915	G/A	BRAF	missense	exon10	c.C1220T	p.P407L
	16:3788614	G/A	CREBBP	missense	exon26	c.C4340T	p.T1447I
	3:38182641	T/C	MYD88	missense	exon5	c.T794C	p.L265P
	6:37138401	G/A	PIM1	missense	exon1	c.G50A	p.C17Y
	6:37139039	C/T	PIM1	stopgain	exon4	c.C379T	p.Q127X
	17:40476822	G/A	STAT3	stopgain	exon17	c.C1507T	p.Q503X
12b	3:38182641	T/C	MYD88	missense	exon5	c.T794C	p.L265P
	6:37138625	C/G	PIM1	stopgain	exon2	c.C159G	p.Y53X
	6:37138946	G/C	PIM1	missense	exon4	c.G286C	p.V96L
	6:37139210	C/T	PIM1	missense	exon4	c.C550T	p.L184F
13	7:2977612	A/T	CARD11	missense	exon8	c.T1072A	p.C358S
	17:62006647	A/G	CD79B	missense	exon6	c.T629C	p.I210T
	17:62006680	A/T	CD79B	missense	exon6	c.T596A	p.L199Q
	3:38182641	T/C	MYD88	missense	exon5	c.T794C	p.L265P
	6:37138808	G/T	PIM1	splice_donor_variant			
	6:37138597	TGGGCAGCGGCG/T	PIM1	frameshift_variant	exon2	c.132del11	p.Leu44fs
	6:37139029	CGAGCCGGT/C	PIM1	frameshift_variant	exon4	c.370delGAGCCGGT	p.Glu124fs
	6:37139210	C/T	PIM1	missense	exon4	c.C550T	p.L184F
	16:81929465	C/T	PLCG2	missense	exon13	c.C1126T	p.R376W
	6:106536324	G/C	PRDM1	missense	exon2	c.G291C	p.E97D
	19:1622393	C/T	TCF3	missense	exon9	c.G571A	p.E191K
14	12:49440169	C/T	KMT2D	missense	exon16	c.G4457A	p.G1486D
	12:49433401	C/T	KMT2D	splice_acceptor_variant	intron31		
	6:37138424	C/G	PIM1	missense	exon1	c.C73G	p.L25V
15	6:37138769	C/T	PIM1	missense	exon3	c.C202T	p.H68Y
	6:37138976	C/T	PIM1	missense	exon4	c.C316T	p.L106F
16	3:38182641	T/C	MYD88	missense	exon5	c.T794C	p.L265P
	6:37138354	G/A	PIM1	start_lost	exon1	c.G3A	p.M1I
	6:37138916	G/C	PIM1	missense	exon4	c.G256C	p.V86L
	6:37139204	C/T	PIM1	missense	exon4	c.C544T	p.L182F

Ref, reference base; Alt, altered base; CDS, coding sequence; aa, aminoacid.

The high frequency of *PIM1* mutations is one of the most relevant findings of this study. We have identified 21 non-synonymous and 20 synonymous *PIM1* mutations in 9 samples. Many of the non-synonymous mutations of *PIM1* affected mainly to the serine/threonine dual specificity protein kinase, catalytic domain (aa 38–290). *MYD88* is the second most frequently mutated gene in this series, and all the cases showed the L265P mutation. The four mutations found in *CD79B* (one case showing 2 mutations) were located in exons 5 and 6 affecting the ITAM domain (aa 185–213), two of them affecting the tyrosine Y196 (Table 3 and Supplementary Table 2).

For one patient (patient number 12), who was diagnosed of PBL on the right breast in 2010 and then of a contralateral relapse in 2015, we sequenced both samples and found that they only shared the L265P-MYD88 mutation and a synonymous one in *PIM1* (Table 3 and Supplementary Table 2). The rest of the mutated genes (*CREBBP, ATM, BRAF* and *STAT3*) and most mutations in *PIM1,* were different. This may suggest that both tumors share a common precursor, but they followed completely different paths of evolution, giving rise to different lymphomas.

## DISCUSSION

In the last twenty years multiple PBL retrospective clinical studies were published including one randomized clinical trial [1–10, 15–16]. However, only two molecular studies, both in oriental population (China and Japan), has been reported [10, 17]. The clinical profile of the patients in this series presented a great deal of similarities with previously published ones (clinical presentation, burden of disease, age and response to treatment), with an average age of 66 years [1–10].

The classification of the cell of origin (COO) by IHC showed that 44% of the samples were GCB, and 33.3% non-GCB. We could not classified 16.6% of them. This results are similar to others series of PB-DLBCL [10, 15, 17–19]. The proliferation index, determined by Ki-67 expression, is strikingly high in these samples, higher than or equal to 80% in 16/18 samples. Previous publications of PBL described the Ki-67 index in a range between 70–90% [9,10, 15, 18, 19].

The high frequency of *PIM1* mutations is one of the most relevant findings of this study. *PIM1* is a gene frequently targeted by aberrant somatic hypermutation [20], but many of the non-synonymous mutations identified in this study were located in the serine/threonine dual specificity protein kinase, catalytic domain (aa 38–290). One of these mutations (p.H68Y) has been previously reported to increase the activity of the enzyme, compared to the wild-type [20]. Others such as those in aa L184, L182, S146 and P125, previously described [21], have been shown not to affect the catalytic activity of the protein. The importance of *PIM1* in the development and evolution of hematologic malignancies, especially in lymphomas, has been known for many years [11, 22–23]. Our results show a higher frequency of mutations in PIM1 (50%) compared with other lymphomas of nodal (12–30%) [13–14] or extra-nodal origin (22–25%) [24–26], which may have implications in the clinical presentation and course of this type of lymphoma. Several studies have explored Pim kinases as a new target for pharmacological inhibition in cancer therapy, including multiple hematologic malignancies and suggest that PB-DLBCL could also benefit by this strategy as demonstrated previously [21, 27–29].

*MYD88* is the second most frequently mutated gene in this series. The seven mutated cases had the L265P mutation, which is the most recurrently found MYD88 mutation, it has been demonstrated to be an activating mutation leading to NFkB activation [30–33] and more frequently found in non-GCB DLBCL [34–35]. The four mutations found in CD79B were in exons 5 and 6 affecting the ITAM domain (aa 185–213). Two of them affected the tyrosine Y196 that was also demonstrated to be a gain-of-function mutation involved in the activation of the BCR- NFkB pathway [31]. Mutations in CARD11 affected the coiled coil and CARD (caspase recruitment) domains and mutations in these domains is a frequent event in DLBCL and have been shown also to activate NFkB in DLBCL cases [31, 36–39].

This is the first study describing a mutational profile of PBL. The study of Taniguchi *et al* [10] was the first to analyze *MYD88* and *CD79B* in a group of 48 patients with lymphomas of the breast. Twenty-eight of them met the diagnostic criteria for PB-DLBCL, according to the authors. Eighteen patients had systemic lymph node involvement or other extra-nodal sites (ovaries, central nervous system, bone marrow, and spleen). The frequencies of the mutations were 58% for *MYD88* and 33% for *CD79B*. These higher frequencies in Taniguchi series could be due to the highest sensitivity of the technique they used, digital polymerase chain reaction, and/or may reflect differences in the prevalence of etiological factors in Spanish and Japanese cohorts. Studies in others extra-nodal lymphomas, such as central nervous system and testis lymphomas, also showed *MYD88, CD79B* and *PIM1* as the most frequently mutated genes, and points out a common molecular profile for extra-nodal DLBCL [24–26]. Recently, a study described the mutational frequency of *MYD88* and *CD79B* in seven PB-DLBCL cases [17]. In this study 5 patients presented mutations in *MYD88* (L265P mutation in 4 cases and 1 case the L265S mutation) and 4 patients presented mutations in *CD79B* (Y196N, Y196H and Y196D). Only 2 of the 7 patients with PB-DLBCL showed mutations in both genes.

**Figure 2:**
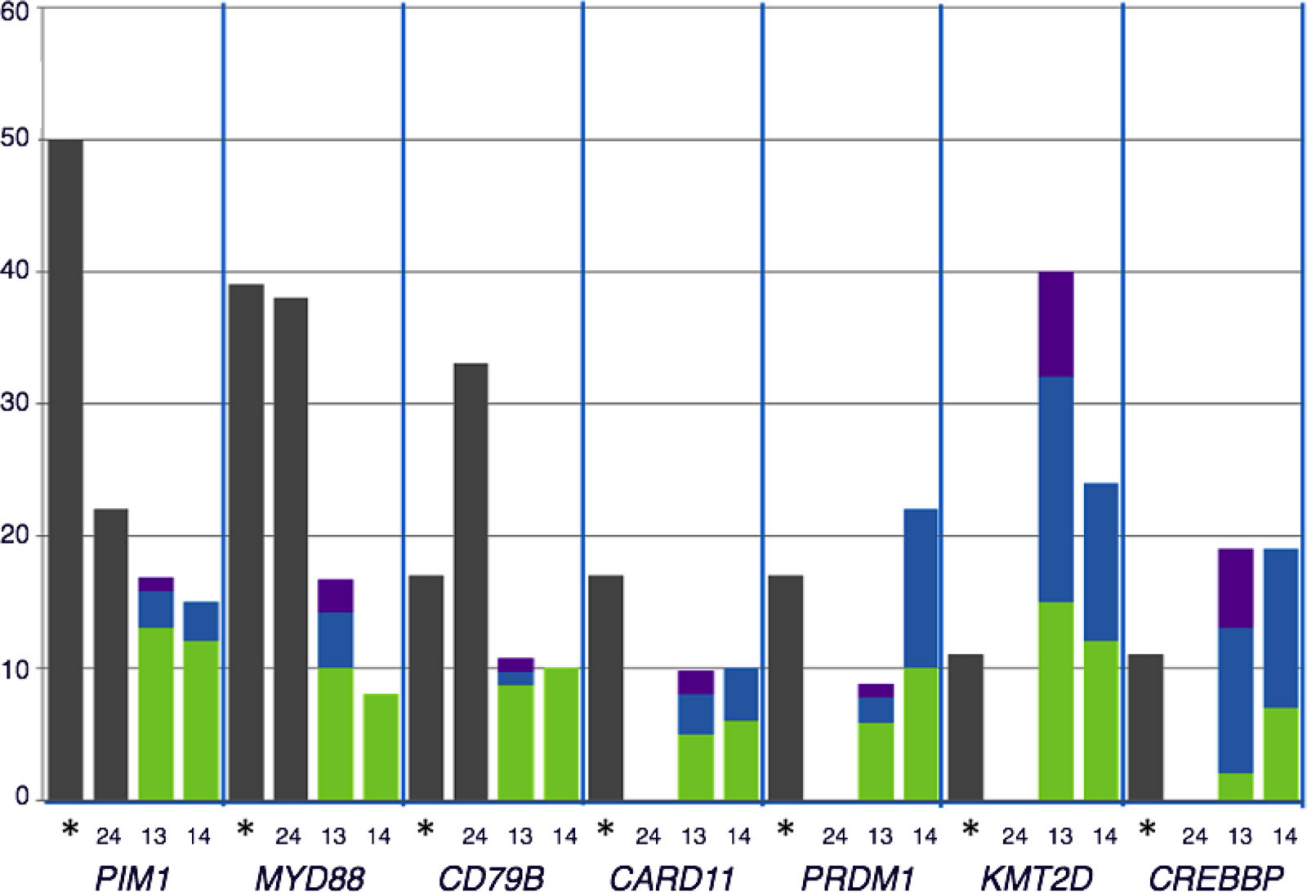
Comparison of the distribution of recurrently mutated genes in DLBCL cohorts: The percentage of mutated cases for each gene represented were obtained from the following data: *our data; [13, 14, 24] The distribution of mutations according to cell of origin (COO) in each series were represented when available. Grey: COO non-classified; green: non-GCB DLBCL, Blue: GCB DLBCL; purple: others

**Figure 3:**
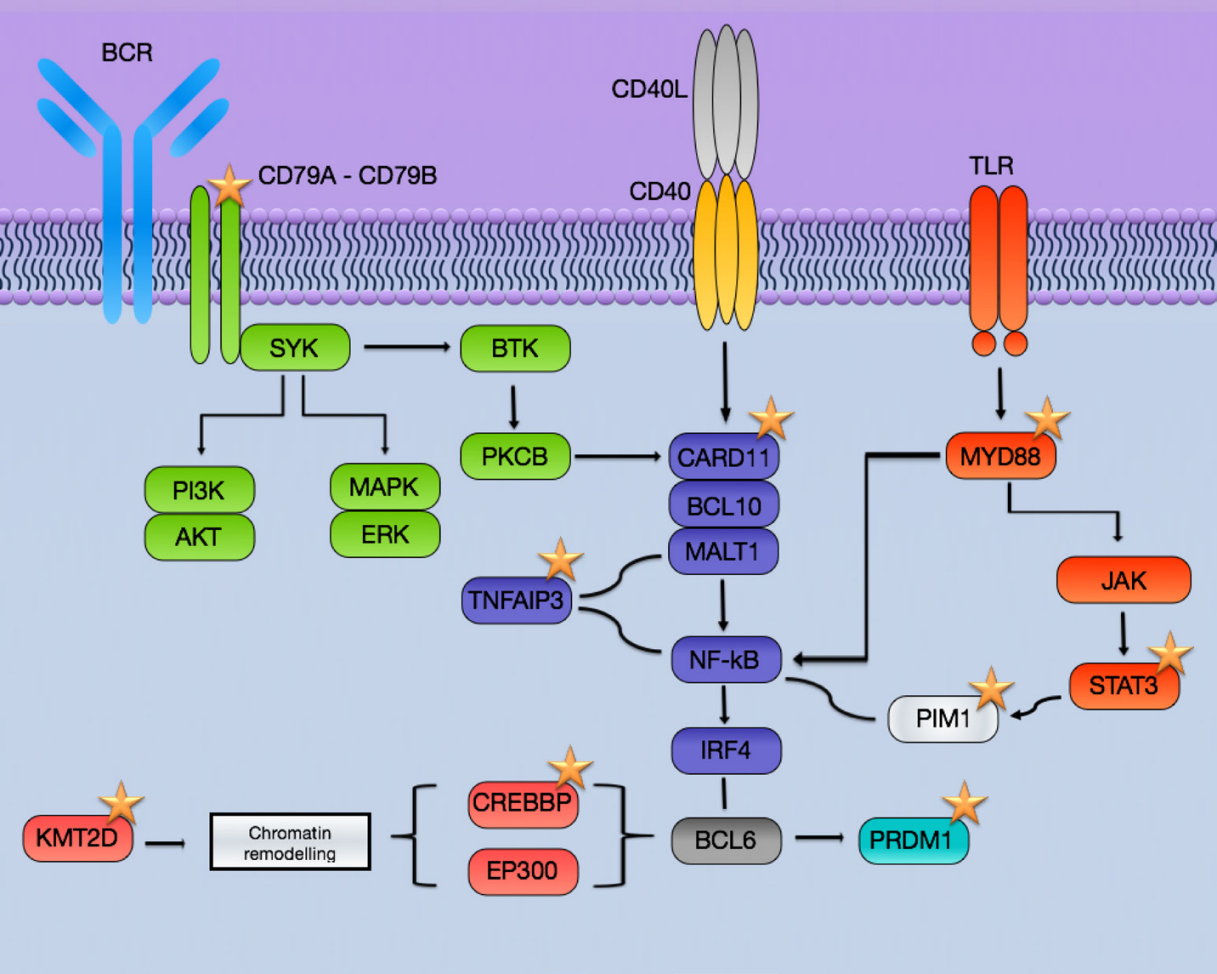
Schematic representation of the pathways affected in PB-DLBCL by mutations The most frequently mutated genes involved BCR and NFkB pathways and are more prevalent in non-GCB DLBCL. *Stars*: recurrently mutated genes in PB-DLBCL.

The most frequently mutated genes (PIM1, MYD88, CD79B, and CARD11) are more prevalent in non-GCB DLBCL as has been addressed in previous publications in both extra-nodal and nodal lymphomas [12, 39–41] (Figure 2). This point out to NFkB as the main pathway targeted for mutations in PB-DLBCL pathogenesis. Activating mutations in CD79B, CARD11 and MYD88 and inactivation mutations in TNFAIP3, leading to activation of NFkB pathway, constitute the hallmark of ABC-like DLBCL as has been reported in multiple publications [34, 35, 40]. This activation of NFkB is a characteristic mainly, although not exclusively, of ABC-DLBCL [13–14, 28–31, 38] (Figure 3). Mutations in other genes such as MLL2/KMT2D and CREBBP have been associated both to GCB and to non-GCB DLBCL [11, 12, 14, 34, 35, 41]. Although, in this series we did not see a clear association between the mutated genes with the COO determined by IHC, several previous studies show that COO classification, using the Hans method, only a variable percentage of cases showed concordance with gene expression profiling classification [42]. In fact, the pattern of mutations in PB-DLBCL seems to converge to NFkB activation, typical of the ABC phenotype. Finally, the presence of altered MYD88, CD79B and PIM1 suggests new therapeutic opportunities for PB-DLBCL using drugs like lenalidomide, BCR, NFkB or PIM inhibitors.

## MATERIALS AND METHODS

### Patients and samples

The study population consists of 17 patients, all women, with PB-DLBCL diagnosed between 1993 and 2016 in different medical centers in Spain. The research project was approved by the Ethics Committee of Puerta de Hierro University Hospital (Majadahonda, Spain) and conducted in accordance with the Declaration of Helsinki. Samples and clinical data were collected, processed and stored according to quality protocols, ensuring the safety and confidentiality of donors’ data. The collected data included sex, age, tumor stage, laterality, treatment, relapse, PFS and OS (Table 1). Clinical data were analyzed according to the modified criteria of Wiseman and Liao [4–5], and the histologic classification of the World Health Organization, 2008. We reviewed the biopsies in a single center to confirm the diagnosis; the COO was determined by IHC data using Hans algorithm.

### Massive parallel sequencing

We designed a targeted sequencing panel for aggressive B-cell lymphomas including 38 recurrently mutated genes described in the literature [11–14] (Supplementary Table 1).

TruSeq^®^ Custom Amplicon Low Input Library for dual-strand sequencing (Illumina Inc. San Diego CA, USA) was used. Dual-strand sequencing eliminates false C-T mutations that can arise from deamination during formalin fixation. The probes for this custom panel were designed with DesignStudio (Illumina) and consisted of 1399 amplicons with an average size of 175 bp and a cumulative targeted region of 140 kb. Polymorphisms were avoided in the design of the primers.

DNA was extracted from formalin fixed and paraffin embedded tissues (FFPET) with truXTRACT FFPE DNA kit (Covaris, Woburn MA USA). Target enrichment was performed according to manufacturer’s instructions. Total amount of input DNA per library ranged from 30 to 100 ng. After library preparation, indexing and bead purification, the libraries (two different libraries per sample, one per strand) were quantified by Qubit (Thermo Fisher Scientific, Waltham, MA, USA) and then normalized with beads and pooled for sequencing. The pooled libraries were sequenced with Miseq Reagent Kit V2 (paired-end, 2x150) on a MiSeq instrument (Illumina), as described in the manufacturer’s protocol.

Median coverage for the amplicons for pool A was 379x (50–1098x) and pool B was 426x (30–2149x). For each sample alignment and variant calling was performed within MiSeq Reporter (Illumina) tool available in the instrument. Additionally, an independent variant calling was done with VarScan 2.4.0 [43] and annotation with Annovar [44]. All the variants identified by both complementary methods were visualized using IGV and only those mutations detected in both pool A and pool B were considered as valid. Data have been deposited in the Sequence Read Archive database (http://www.ncbi.nlm.nih.gov/sra) (SRP119626).

## SUPPLEMENTARY MATERIALS TABLES



## Abbreviations

COO: Cell of origin
CR: complete response
DLBCL: diffuse large B-cell lymphoma
FFPET: formalin fixed and paraffin embedded tissues
GCB: germinal center B-cell
NHL: non-Hodgkin’s lymphomas
OS: overall survival
PBL: Primary breast lymphoma
PB-DLBCL: primary breast diffuse large B-cell lymphoma
PFS: progression free survival
R-CHOP: Cyclophosphamide, Doxorrubicin, Vincristine, Prednisone and Rituximab

## References

[1] Validire P, Capovilla C, Asselain B, Kirova Y, Goudefroye R, Plancher C, Fourquet A, Zanni M, Gaulard P, Vicente-Salomon A, Decaudin D (2009). Primary breast non-Hodgkin’s lymphoma: a large single center study of initial characteristics, natural history, and prognostic factors. Am J Hematol.

[2] Aviv A, Tadmor T, Polliack A (2013). Primary diffuse large B-cell lymphoma of the breast: looking at pathogenesis, clinical issues and therapeutic options. Ann Oncol.

[3] Ryan G, Martinelli G, Kuper-Hommel M, Tsang R, Pruneri G, Yuen K, Roos D, Lennard A, Devizzi L, Crabb S, Hossfeld D, Pratt G, Dell’Olio M (2008). Primary diffuse large B-cell lymphoma of the breast: prognostic factors and outcomes of a study by the International Extranodal Lymphoma Study Group. Ann Oncol.

[4] Wiseman C, Liao K (1972). Primary lymphoma of the breast. Cancer.

[5] Hugh JC, Jackson F, Hanson J, Poppema S (1990). Primary breast lymphoma. An immunohistologic study of 20 new cases. Cancer.

[6] Franco F, Lavernia J, Aguiar-Bujanda D, Miramón J, Gumá J, Álvarez R, Gómez-Codina J, Arroyo FG, Llanos M, Marín M, Alfaro J, Quero C, Delgado M (2016). Primary Breast Lymphoma: Analysis of 55 Cases of the Spanish Lymphoma Oncology Group. Clin Lymphoma, Myeloma Leuk.

[7] Cheah CY, Campbell B, Seymour J (2014). Primary breast lymphoma. Cancer Treat Rev.

[8] Niitsu N, Okamoto M, Nakamine H, Hirano M (2008). Clinicopathologic features and treatment outcome of primary breast diffuse large B-cell lymphoma. Leuk Res.

[9] Hosein P, Maragulia J, Salzberg M, Press OW, Habermann TM, Vose JM, Bast M, Advani RH, Tibshirani R, Evens AM, Islam N, Leonard JP, Martin P (2014). A multicentre study of primary breast diffuse large B-cell lymphoma in the rituximab era. Br J Haematol.

[10] Taniguchi K, Katsuyoshi T, Chuang S, Miyata-Takata T, Sato Y, Satou A, Hashimoto Y, Tamura M, Nagakita K, Ohnishi N, Noujima-Harada M, Tabata T, Kikuti YY (2016). Frequent MYD88 L265P and CD79B mutations in primary breast diffuse large b-cell lymphoma. Am J Surg Pathol.

[11] Zhang J, Grubor V, Love CL, Banerjee A, Richards KL, Mieczkowski PA, Dunphy C, Choi W, Au WY, Srivastana G, Lugar PL, Rizzieri DA, Lagoos AS (2013). Genetic heterogeneity of diffuse large B-cell lymphoma. PNAS.

[12] Basso K, Dalla-Favera R (2015). Germinal centres and B cell lymphomagenesis. Nat Rev Immunol.

[13] Dubois S, Viailly PJ, Mareschal S, Bohers E, Bertrand P, Ruminy P, Maingonnat C, Jais JP, Peyrouze P, Figeac M, Molina TJ, Desmots F, Fest T (2016). Next-Generation Sequencing in Diffuse Large B-Cell Lymphoma Highlights Molecular Divergence and Therapeutic Opportunities: a LYSA Study. Clin Cancer Res.

[14] Pasqualucci L, Trifonov V, Fabbri G, Ma J, Rossi D, Chiarenza A, Wells VA, Grunn A, Messina M, Elliot O, Chan J, Bhagat G, Chadburn A (2011). Analysis of the coding genome of diffuse large B-cell lymphoma. Nat Genet.

[15] Avilés A, Neri N, Nambo J (2012). The role of genotype in 104 cases of diffuse large b-cell lymphoma primary of breast. Am J Clin Oncol.

[16] Avilés A, Delgado S, Nambo MJ, Neri N, Murillo E, Cleto S (2005). Primary breast lymphoma: results of a controlled clinical trial. Oncology.

[17] Cao XX, Li J, Cai H, Zhang W, Duan MH, Zhou DB (2017). Patients with primary breast and primary female genital tract diffuse large B cell lymphoma have a high frequency of MYD88 and CD79B mutations. Ann Hematol.

[18] Talwalkar S, Miranda R, Valbuena J, Routbort M, Martin A, Medeiros L (2008). Lymphomas involving the breast. a study of 106 cases comparing localized and disseminated neoplasms. Am J Surg Pathol.

[19] Li D, Deng J, He H, Bu Y, Peng F, Tansg X, Wang B, Lei Y, Zhang H, Xie P (2012). Primary breast diffuse large B-cell lymphoma shows an activated B-cell–like phenotype. Ann Diagn Pathol.

[20] Kumar A, Mandiyan V, Suzuki Y, Zhang C, Rice J, Tsai J, Artis DR, Ibrahim P, Bremer R (2005). Crystal structures of proto-oncogene kinase Pim1: a target of aberrant somatic hypermutations in diffuse large cell lymphoma. J Mol Biol.

[21] Peters TL, Li L, Tula-Sanchez AA, Pongtornpipat P, Schatz JH (2016). Control of translational activation by PIM kinase in activated B-cell diffuse large B-cell lymphoma confers sensitivity to inhibition by PIM447. Oncotarget.

[22] Mondello P, Cuzzocrea S, Mian M (2014). Pim kinases in hematological malignancies: where are we now and where are we going?. J Hematol Oncol.

[23] Nawijn M, Alendar A, Berns A (2011). For better or for worse: the role of Pim oncogenes in tumorigenesis. Nat Rev Cancer.

[24] Bruno A, Boisselier B, Labreche K, Marie Y, Polivka M, Jouvet A, Adam C, Figarella-Branger D, Miquel C, Eimer S, Houillier C, Soussain C, Mokhtari K (2014). Mutational analysis of primary central nervous system lymphoma. Oncotarget.

[25] Nakamura T, Tateishi K, Niwa T, Matsushita Y, Tamura K, Kinoshita M, Tanaka K, Fulushima S, Takami H, Arita H, Kubo A, Shuto T, Ohno M (2016). Recurrent mutations of CD79B and MYD88 are the hallmark of primary central nervous system lymphomas. Neuropathol Appl Neurobiol.

[26] Cao Y, Zhu T, Zhang P, Xiao M, Yi S, Yang Y, Li Q, Ling S, Wang Y, Gao L, Zhu L, Wang J, Wang N (2016). Mutations or copy number losses of CD58 and TP53 genes in diffuse large B cell lymphoma are independent unfavorable prognostic factors. Oncotarget.

[27] Szydłowski M, Prochorec-Sobieszek M, Szumera-Cieckiewicz A, Derezinska E, Hoser G, Wasilewska D, Szymańska-Giemza O, Jabłońska E, Białopiotrowicz E, Sewastianik T, Polak A, Czardybon W, Gałęzowski M (2017). Expression of PIM kinases in Reed-Sternberg cells fosters immune privilege and tumor cell survival in Hodgkin lymphoma. Blood.

[28] Martín-Sanchez E, Odqvist L, Rodríguez-Pinilla SM, Sánchez-Beato M, Roncador G, Domínguez-González B, Blanco-Aparicio C, García Collazo AM, Cantalapiedra EG, Fernández JP, Curiel del Olmo S, Pisonero H, Madureira R (2014). PIM kinases as potential therapeutic targets in a subset of peripheral T cell lymphoma cases. PLoS One.

[29] Gómez-Abad C, Pisonero H, Blanco-Aparicio C, Roncador G, González-Menchén A, Martinez-Climent JA, Mata E, Rodriguez ME, Muñóz-Gonzalez G, Sánchez-Beato M, Leal JF, Bischoff JR, Piris MA (2011). PIM2 inhibition as a rational therapeutic approach in B-cell lymphoma. Blood.

[30] Ngo VN, Young RM, Schmitz R, Jhavar S, Xiao W, Lim KH, Kohlhammer H, Xu W, Yang Y, Zhao H, Shaffer AL, Romesser P, Wright G (2011). Oncogenically active MYD88 mutations in human lymphoma. Nature.

[31] Davis RE, Ngo VN, Lenz G, Tolar P, Young RM, Romesser PB, Kohlhammer H, Lamy L, Zhao H, Yang Ym, Xu W, Shaffer AL, Wright G (2010). Chronic active B-cell-receptor signalling in diffuse large B-cell lymphoma. Nature.

[32] Zheng M, Perry AM, Bierman P, Loberiza F, Nasr MR, Szwajcer D, Del Bigio MR, Smith LM, Zhang W, Greiner TC (2017). Frequency of MYD88 and CD79B mutations, and MGMT methylation in primary central nervous system diffuse large B-cell lymphoma. Neuropathology.

[33] Lee JH, Jeong H, Choi JW, Oh H, Kim YS (2017). Clinicopathologic significance of MYD88 L265P mutation in diffuse large B-cell lymphoma: a meta-analysis. Sci Rep.

[34] Lenz G, Davis RE, Ngo VN, Lam L, George TC, Wright GW, Dave SS, Zhao Hm, Xu W, Rosenwald A, Ott G, Muller-Hermelink HK, Gascoyne RD (2008). Oncogenic CARD11 mutations in human diffuse large B cell lymphoma. Science.

[35] Takahara T, Matsuo K, Seto M, Nakamura S, Tsuzuki S (2016). Synergistic activity of Card11 mutant and Bcl6 in the development of diffuse large B-cell lymphoma in a mouse model. Cancer Sci.

[36] Knittel G, Liedgens P, Korovkina D, Pallasch CP, Reinhardt HC (2016). Rewired NFκB signaling as a potentially actionable feature of activated B-cell-like diffuse large B-cell lymphoma. Eur J Haematol.

[37] Bu R, Bavi P, Abubaker J, Jehan Z, Al-haqawi W, Ajarim D, Al-Dayel F, Uddin S, Al-Kuraya KS (2012). Role of nuclear factor-κB regulators TNFAIP3 and CARD11 in Middle Eastern diffuse large B-cell lymphoma. Leuk Lymphoma.

[38] Odqvist L, Montes-Moreno S, Sánchez-Pacheco RE, Young KH, Martín-Sánchez E, Cereceda L, Sánchez-Verde L, Pajares R, Mollejo M, Fresno MF, Mazorra F, Ruíz-Marcellán C, Sánchez-Beato M (2014). NFκB expression is a feature of both activated B-cell-like and germinal center B-cell-like subtypes of diffuse large B-cell lymphoma. Mod Pathol.

[39] Young RM, Staudt LM (2013). Targeting pathological B cell receptor signalling in lymphoid malignancies. Nat Rev Drug Discov.

[40] Dalla-Favera R (2017). Molecular genetics of aggressive B-cell lymphoma. Hematol Oncol.

[41] Juskevicius D, Jucker D, Klingbiel D, Mamot C, Dirnhofer S, Tzankov A (2017). Mutations of CREBBP and SOCS1 are independent prognostic factors in diffuse large B celllymphoma: mutational analysis of the SAKK 38/07 prospective clinical trial cohort. J Hematol Oncol.

[42] Gutiérrez-García G, Cardesa-Salzmann T, Climent F, González-Barca E, Mercadal S, Mate JL, Sancho JM, Arenillas L, Serrano S, Escoda L, Martínez S, Valera A, Martínez A (2011). Gene-expression profiling and not immunophenotypic algorithms predicts prognosis in patients with diffuse large B-cell lymphoma treated with immunochemotherapy. Blood.

[43] Koboldt DC, Zhang Q, Larson DE, Shen D, McLellan MD, Lin L, Miller CA, Mardis ER, Ding L, Wilson RK (2012). VarScan 2: somatic mutation and copy number alteration discovery in cancer by exome sequencing. Genome Res.

[44] Wang K, Li M, Hakonarson H. ANNOVAR (2010). functional annotation of genetic variants from high-throughput sequencing data. Nucleic Acids Res.

